# False Positive Cushing's Syndrome in a 16-Year-Old Male

**DOI:** 10.5812/ijem-158085

**Published:** 2025-01-30

**Authors:** Mahdi Paksaz, Hedieh Saneifard, Alimohammad Mirdehghan, Marjan Shakiba, Asieh Mosallanejad

**Affiliations:** 1 Student Research Center, Faculty of Medicine, Shahid Beheshti University of Medical Sciences, Tehran, Iran; 2 Department of Pediatric Endocrinology and Metabolism, Mofid Children's Hospital, Shahid Beheshti University of Medical Sciences, Tehran, Iran; 3 Shahid Beheshti University of Medical Sciences, Tehran, Iran

**Keywords:** Cushing’s Syndrome, Dexamethasone Suppression Test, False-Positive Diagnosis, Adolescent Weight Gain

## Abstract

**Introduction:**

Cushing syndrome is an endocrine disorder characterized by prolonged exposure to high levels of glucocorticoids, either from endogenous overproduction or exogenous sources. It presents with symptoms such as rapid weight gain, central obesity, muscle weakness, and hypertension. The diagnosis requires a combination of clinical, biochemical, and imaging tests. Dexamethasone suppression testing is pivotal in diagnosing hypercortisolism, but its accuracy may be affected by pharmacokinetic factors, such as drug interactions. This case report discusses a false-positive diagnosis of Cushing syndrome in a 16-year-old male, caused by the pharmacokinetic interference of carbamazepine, an enzyme-inducing medication.

**Case Presentation:**

A 16-year-old male with psychiatric comorbidities (bipolar disorder and epilepsy) presented with rapid weight gain, a hallmark symptom of Cushing syndrome. Initial laboratory testing revealed elevated cortisol and adrenocorticotropic hormone (ACTH) levels, with partial cortisol suppression during the low-dose dexamethasone suppression test, suggesting an ACTH-dependent cause of hypercortisolism. Imaging studies of the pituitary and adrenal glands were negative for abnormalities. High-dose dexamethasone suppression and 24-hour urinary free cortisol (UFC) levels further supported the diagnosis of hypercortisolism. A detailed medication review revealed the patient was taking carbamazepine, a CYP3A4 enzyme inducer, which could have accelerated the metabolism of dexamethasone and led to inadequate suppression of cortisol, producing falsely elevated levels.

**Conclusions:**

Carbamazepine, through its enzyme-inducing effect on CYP3A4, likely interfered with the dexamethasone suppression test, leading to false-positive results for Cushing syndrome. Following the discontinuation of carbamazepine, the patient’s cortisol levels normalized, weight stabilized, and the signs of Cushing syndrome resolved. Alternative psychiatric medications were initiated without further endocrine abnormalities. This case emphasizes the importance of considering pharmacokinetic interactions, such as those with enzyme-inducing drugs, when diagnosing Cushing syndrome. Clinicians should carefully review medications in patients with suspected hypercortisolism and consider these interactions when interpreting biochemical test results. A comprehensive medication review and interdisciplinary collaboration are crucial for accurate diagnosis, avoiding unnecessary interventions, and improving patient outcomes. The case advocates for tailored diagnostic protocols in similar clinical scenarios.

## 1. Introduction

Cushing syndrome is an endocrine disorder caused by prolonged exposure to elevated levels of glucocorticoids, which can result from either endogenous overproduction or exogenous sources. Characterized by symptoms such as rapid weight gain, central obesity, muscle weakness, and hypertension, it is clinically significant due to its wide-ranging effects on metabolic, cardiovascular, and immune health (1-3). Cushing syndrome is traditionally divided into two types: adrenocorticotropic hormone (ACTH)-dependent, often caused by pituitary adenomas, and ACTH-independent, arising from adrenal pathologies or ectopic ACTH. Diagnosis of Cushing syndrome is complex, requiring a combination of clinical assessment, biochemical tests, and imaging studies to determine the source and severity of hypercortisolism (4, 5).

One of the principal diagnostic tools in evaluating suspected Cushing syndrome is the dexamethasone suppression test. In this test, a synthetic glucocorticoid, dexamethasone, is administered, and subsequent cortisol levels are measured to assess the body's response. Under normal conditions, dexamethasone administration suppresses cortisol production through feedback inhibition of the hypothalamic-pituitary-adrenal (HPA) axis. In individuals with Cushing syndrome, however, this feedback mechanism is disrupted, leading to persistently elevated cortisol levels despite dexamethasone administration. If the overnight dexamethasone test is positive, complementary tests, including low-dose and high-dose dexamethasone suppression tests, help differentiate between pituitary, adrenal, and ectopic sources of ACTH.

While dexamethasone suppression testing is generally reliable, several factors can influence test accuracy, including pharmacological interactions that modify dexamethasone metabolism (6, 7). Medications that induce hepatic cytochrome P450 enzymes, particularly CYP3A4, are known to accelerate dexamethasone metabolism, potentially leading to reduced blood levels of the drug and altering test outcomes. Antiepileptic drugs (AEDs) such as carbamazepine and phenytoin are potent inducers of CYP3A4 and have been documented to increase the metabolism of glucocorticoids, including dexamethasone. This pharmacokinetic interaction can result in a rapid clearance of dexamethasone from circulation, potentially leading to false-positive results in dexamethasone suppression tests. In clinical practice, this presents a challenge, as patients receiving AEDs may exhibit cortisol levels that are suggestive of Cushing syndrome despite a lack of true hypercortisolism, thereby confounding the diagnostic process (8, 9). This case report presents a unique example of diagnostic complexity in a 16-year-old male with rapid weight gain initially suspected to be Cushing syndrome.

## 2. Case Presentation

The patient is a 16-year-old male with a history of psychiatric illness, who presented with substantial weight gain, raising concerns for possible Cushing syndrome. His weight gain had been rapid and excessive, with approximately 30 kg added over 5 months. At the time of evaluation, his height was 183 cm, and his weight was 120 kg (BMI = 35.8 kg/m²). The patient’s family history revealed a tendency towards weight gain, but no cases of endocrine disorders were reported. The patient had been under psychiatric care for the past 8 months for mood stabilization and other psychiatric symptoms, which were being managed through a regimen of psychotropic and antiepileptic medications.

In October 2023, the patient began treatment with a combination of medications, including carbamazepine 200 mg twice daily, lithium 300 mg three times daily, haloperidol 0.5 mg twice daily, topiramate 200 mg daily in two doses, valproate (Depakene) 300 mg in three doses, and biperiden 2 mg twice daily, as part of his psychiatric management. Shortly after starting this treatment, the patient’s weight gain accelerated, alongside other symptoms often associated with Cushing syndrome, such as fatigue and altered body composition. Importantly, he had no history of corticosteroid use, which could have otherwise contributed to exogenous Cushing syndrome.

On physical examination, the patient appeared older than his stated age, with signs of central obesity. There was noticeable truncal obesity with a round face, and the neck showed signs of fat accumulation. The skin was thin, and there were visible striae on the abdomen and thighs, which were purple and wide. The chest examination showed a slightly increased upper body fat distribution, but no visible signs of gynecomastia. Cardiovascular examination revealed normal heart sounds, intact and symmetrical peripheral pulses, and no edema in extremities. The patient had mild hypertension, with blood pressure recorded at 135/85 mmHg. There was central obesity with a pendulous abdomen, no hepatomegaly, or tenderness to palpation. Striae were visible across the abdomen and thighs. Clear breath sounds were auscultated bilaterally with no wheezing or crackles. The patient demonstrated mild proximal muscle weakness, especially in the shoulders and hips. There were no signs of joint deformity or swelling. Gait was normal, though the patient reported mild difficulty standing from a seated position due to muscle weakness. Normal testicular size and no abnormalities were noted in the genital examination.

Due to the rapid and disproportionate weight gain, an endocrine workup was initiated, beginning with assessments of cortisol and ACTH levels. Baseline morning cortisol was elevated at 25 µg/dL (reference range: 6.2 - 20 µg/dL), with an ACTH level of 19.26 pg/mL (reference range: 7.1 - 56.3 pg/mL), raising suspicion for Cushing’s syndrome. A dexamethasone suppression test was subsequently ordered to confirm the diagnosis. The patient underwent a 1 mg overnight dexamethasone suppression test, and the follow-up serum cortisol level was measured at 6.68 µg/dL, exceeding the threshold of < 1.8 µg/dL. The partial suppression of cortisol suggested the possibility of endogenous Cushing’s syndrome.

Given the initial positive result, a series of tests were conducted to determine the source of hypercortisolism. The 24-hour urinary free cortisol (UFC) measured 260 µg/day, exceeding the laboratory reference range of 36 - 137 µg/day. The normal range for 24-hour UFC is less than 10 µg/day. The patient then underwent a low-dose dexamethasone suppression test, receiving 0.5 mg every 6 hours for 48 hours. After this regimen, the serum cortisol level was measured at 7 µg/dL, indicating persistent cortisol secretion. The normal range for serum cortisol is less than 1.8 µg/dL. Following this, a high-dose dexamethasone suppression test was performed, which again demonstrated inadequate suppression, with cortisol levels of 8.3 µg/dL and ACTH at 21 pg/mL (reference range: 7.1 - 56.3 pg/mL). These results continued to support a diagnosis of ACTH-dependent Cushing’s syndrome. Additionally, a subsequent UFC test revealed a level of 270 µg/day (laboratory reference range: 36 - 137 µg/day), indicating sustained cortisol secretion without suppression from the baseline.

An MRI of the pituitary gland was performed at another center, revealing no abnormalities, thereby ruling out the presence of a pituitary adenoma. Given the persistence of symptoms and diagnostic uncertainty, the patient was admitted for inpatient evaluation. During this admission, a high-frequency dexamethasone suppression test was conducted, with blood sampling every 15 minutes to monitor cortisol and ACTH responses (results detailed in Table 1). 

**Table 1.** A High-Frequency Dexamethasone Suppression Test Was Conducted with Blood Sampling Every 15 Minutes to Monitor Cortisol and Adrenocorticotropic Hormone Responses

Variables	Time (min)					
	-15	0	+15	+30	+45	+60
**Cortisol (µg/dL)**	7.48	8.14	9.58	9.18	9.89	10.3
**ACTH (pg/mL)**	17	8.4	21.8	21.3	23.4	19.1

Abbreviation: ACTH, adrenocorticotropic hormone.

The lack of significant cortisol suppression, despite intensive monitoring and dexamethasone administration, sustained the suspicion of an ACTH-dependent mechanism. However, with no pituitary abnormalities, inferior petrosal sinus sampling (IPSS) was ordered to further localize ACTH. During the course of his endocrine evaluation, a thorough medication review was conducted, and the impact of his psychiatric medications on diagnostic testing was considered. Carbamazepine, in particular, was identified as a potent inducer of cytochrome P450 enzymes, specifically CYP3A4, which is known to accelerate the metabolism of dexamethasone (10). Given this pharmacokinetic interaction, it was hypothesized that carbamazepine might have increased dexamethasone clearance, thereby producing an artificially low blood level during suppression tests. This would result in a falsely elevated cortisol level, mimicking the appearance of Cushing syndrome.

Based on this insight, carbamazepine and valproate (Depakine) were discontinued in early September 2024. Following the discontinuation of these drugs, the patient’s weight gain plateaued, and his rapid increase in body mass ceased. No additional symptoms of Cushing syndrome developed, and subsequent evaluations showed no further abnormalities in cortisol levels (results detailed in Table 2). This clinical outcome strongly supported the hypothesis that carbamazepine had been inducing a false-positive result on dexamethasone suppression tests due to accelerated glucocorticoid metabolism.

**Table 2.** Serum Cortisol Level After Carbamazepine and Valproate (Depakine) Were Discontinued

Variable	Before Getting Dexamethasone	After Getting Dexamethasone	Reference Range
**Serum cortisol (µg/dL)**	15.1	< 0.8	< 1.8

Since the cessation of carbamazepine and valproate, the patient’s psychiatric management has been adjusted to avoid medications that could interfere with cortisol metabolism. He was transitioned to chlorpromazine (25 mg), aripiprazole (10 mg daily), biperiden (1 mg daily), and lithium. His weight has remained stable, and no further testing indicated Cushing syndrome. The patient is under ongoing monitoring with periodic endocrinological follow-up to assess metabolic health and ensure stability in his psychiatric symptoms.

## 3. Discussion

The pharmacokinetic interaction between carbamazepine and dexamethasone is central to this case and has significant implications for endocrine diagnostics. Carbamazepine, a widely used antiepileptic and mood-stabilizing medication, is a potent inducer of hepatic cytochrome P450 enzymes, particularly CYP3A4. This enzyme plays a critical role in metabolizing various drugs, including corticosteroids like dexamethasone. When a patient takes carbamazepine, CYP3A4 enzyme activity increases, leading to enhanced metabolism and clearance of dexamethasone from the bloodstream. As dexamethasone levels drop more rapidly than expected, this can affect diagnostic results, as seen in this case where standard suppression doses failed to maintain sufficient blood levels of dexamethasone to suppress cortisol production. This pharmacokinetic acceleration results in falsely elevated cortisol readings on suppression tests, mimicking a Cushing syndrome profile in patients who do not have this disorder (10-12).

The effects of this metabolic interaction are well-documented in cases where carbamazepine reduces the therapeutic efficacy of other medications metabolized through the CYP3A4 pathway. However, this case report underscores an often-overlooked consequence of this interaction within the context of endocrine diagnostics (10, 11). Since dexamethasone is commonly used to evaluate suspected Cushing syndrome, understanding the implications of carbamazepine and similar enzyme-inducing drugs on test results is essential for accurate diagnosis (13, 14).

The false-positive results in this patient led to an extensive and costly workup. Had the interaction not been identified, the patient would have undergone additional testing and possibly invasive procedures, such as IPSS or even exploratory surgeries. Moreover, this diagnostic ambiguity could have resulted in an incorrect diagnosis of Cushing syndrome and subsequent unnecessary treatments, including possible surgery or glucocorticoid-suppressing medications. This highlights the need for increased awareness among clinicians regarding potential drug interactions, especially in patients who present with unusual symptoms while on medications that impact metabolic enzymes.

While many reports address therapeutic failures due to the interaction between AEDs and corticosteroids, relatively few focus on diagnostic consequences, especially in endocrinology. Our case contributes to the literature by illustrating how such interactions can create false-positive results on suppression tests. This case suggests that clinicians should approach dexamethasone suppression test results cautiously in patients on AEDs, particularly carbamazepine, as standard test interpretations may not apply.

Purple or red abdominal striae, commonly referred to as stretch marks, are a prominent physical feature frequently linked to Cushing syndrome. However, they may also appear in pregnant women, individuals with obesity, and adolescents (15). These striae present as linear, smooth bands of skin that appear atrophic, transitioning in color from red to purple and eventually to white (16). The diagnostic management discussed has helped exclude Cushing syndrome.

Among the various biochemical tests used to evaluate Cushing syndrome, UFC is considered one of the most feasible options (17). A significant challenge of using UFC as a diagnostic screening test is its low specificity. It has also been suggested that elevated UFC excretion may not consistently occur in pediatric cases of Cushing syndrome, complicating the ability to rule out the diagnosis (18). Numerous medical conditions cause physiological stress, leading to an overproduction of cortisol and elevated UFC levels. This is commonly observed in patients with hypertension. Patients with overt obesity or diabetes may also exhibit elevated UFC levels, leading to false-positive test results (19). Based on the discussed paraclinical findings, along with the supportive medical history and examination data, the elevated UFC level in this patient is not attributed to Cushing syndrome.

### 3.1. Recommendations for Clinical Practice

The insights gained from this case can help inform clinical guidelines for managing suspected Cushing’s syndrome in patients taking carbamazepine or similar enzyme-inducing drugs. To improve diagnostic accuracy, we recommend the following strategies:

#### 3.1.1. Medication Review Prior to Suppression Testing

A comprehensive medication history, including potential enzyme inducers, should be mandatory before initiating dexamethasone suppression tests. Clinicians should consider the possibility of rapid dexamethasone metabolism in patients taking AEDs or other drugs known to induce CYP3A4.

#### 3.1.2. Alternative Testing Approaches

When suppression tests are warranted in patients on enzyme-inducing drugs, clinicians may consider alternative testing protocols. Options include using higher doses of dexamethasone, extending the suppression period, or exploring alternative diagnostic modalities such as midnight salivary cortisol or UFC, which may be less affected by drug interactions.

#### 3.1.3. Future Research Directions

Additional studies are needed to quantify the exact degree of dexamethasone metabolism induced by carbamazepine and other AEDs. This would allow clinicians to adjust dexamethasone doses more precisely and develop standardized protocols for interpreting test results in these patients. Further research should also explore alternative diagnostic approaches for Cushing’s syndrome in patients requiring enzyme-inducing medications.

### 3.2. Conclusions

This case report demonstrates the diagnostic complexities associated with pharmacokinetic interactions between carbamazepine and dexamethasone in a young male presenting with rapid weight gain. The false-positive suppression test results due to accelerated dexamethasone metabolism highlight the importance of comprehensive medication assessments in endocrinology, especially when interpreting suppression test results. Interdisciplinary collaboration and awareness of potential pharmacologic interferences are essential for accurate diagnosis and management. By sharing this case, we hope to underscore the need for careful consideration of drug interactions in endocrine diagnostics, ultimately improving patient care and reducing the risk of misdiagnosis.

### 3.3. Limitations

While assessing serum dexamethasone levels could help reduce the need for additional paraclinical tests, this test is unfortunately not widely accessible in Iran, specifically in this center. Consequently, the clinical and paraclinical approach outlined in this article is influenced by this limitation.

## Data Availability

The dataset presented in the study is available on request from the corresponding author during submission or after publication. The data are not publicly available due to the patient's privacy policies of the mofid childrens hospital.
